# Has Epizootic Become Enzootic? Evidence for a Fundamental Change in the Infection Dynamics of Highly Pathogenic Avian Influenza in Europe, 2021

**DOI:** 10.1128/mbio.00609-22

**Published:** 2022-06-21

**Authors:** Anne Pohlmann, Jacqueline King, Alice Fusaro, Bianca Zecchin, Ashley C. Banyard, Ian H. Brown, Alexander M. P. Byrne, Nancy Beerens, Yuan Liang, Rene Heutink, Frank Harders, Joe James, Scott M. Reid, Rowena D. E. Hansen, Nicola S. Lewis, Charlotte Hjulsager, Lars E. Larsen, Siamak Zohari, Kristofer Anderson, Caroline Bröjer, Alexander Nagy, Vladimir Savič, Steven van Borm, Mieke Steensels, Francois-Xavier Briand, Edyta Swieton, Krzysztof Smietanka, Christian Grund, Martin Beer, Timm Harder

**Affiliations:** a Institute of Diagnostic Virology, Friedrich Loeffler Institute, Greifswald-Insel Riems, Germany; b European Union Reference Laboratory for Avian Influenza and Newcastle Disease, Istituto Zooprofilattico Sperimentale delle Venezie, Legnaro, Padua, Italy; c Animal and Plant Health Agency —Weybridge, New Haw, Addlestone, Surrey, United Kingdom; d OIE/FAO International Reference Laboratory for Avian Influenza, Swine Influenza and Newcastle Disease, Animal and Plant Health Agency —Weybridge, Addlestone, Surrey, United Kingdom; e Wageningen Bioveterinary Research, Lelystad, The Netherlands; f Department of Veterinary and Animal Sciences, University of Copenhagen, Frederiksberg, Denmark; g Department of Pathobiology and Population Sciences, Royal Veterinary College, Hatfield, United Kingdom; h Department for Virus and Microbiological Special Diagnostics, Statens Serum Institut, Copenhagen, Denmark; i Department of Microbiology, National Veterinary Institute, SVA, Uppsala, Sweden; j Department of Pathology, National Veterinary Institute, SVA, Uppsala, Sweden; k State Veterinary Institute Prague, Prague, Czech Republic; l Croatian Veterinary Institute, Poultry Centre, Zagreb, Croatia; m Service of Avian Virology and Immunology, Sciensano, Brussels, Belgium; n Agence Nationale de Sécurité Sanitaire, de l’Alimentation, de l’Environnement et du Travail, Laboratoire de Ploufragan-Plouzané-Niort, Unité de Virologie, Immunologie, Parasitologie Avaires et Cunicoles, Ploufragan, France; o Department of Poultry Diseases, National Veterinary Research Institute, Puławy, Poland; University of Hong Kong

**Keywords:** high-pathogenicity avian influenza, migratory birds, poultry, enzootic, evolution, Europe, enzootic evolution

## Abstract

Phylogenetic evidence from the recent resurgence of high-pathogenicity avian influenza (HPAI) virus subtype H5N1, clade 2.3.4.4b, observed in European wild birds and poultry since October 2021, suggests at least two different and distinct reservoirs. We propose contrasting hypotheses for this emergence: (i) resident viruses have been maintained, presumably in wild birds, in northern Europe throughout the summer of 2021 to cause some of the outbreaks that are part of the most recent autumn/winter 2021 epizootic, or (ii) further virus variants were reintroduced by migratory birds, and these two sources of reintroduction have driven the HPAI resurgence. Viruses from these two principal sources can be distinguished by their hemagglutinin genes, which segregate into two distinct sublineages (termed B1 and B2) within clade 2.3.4.4b, as well as their different internal gene compositions. The evidence of enzootic HPAI virus circulation during the summer of 2021 indicates a possible paradigm shift in the epidemiology of HPAI in Europe.

## OPINION/HYPOTHESIS

## RECENT EPIZOOTIC EVENTS REGARDING HIGHLY PATHOGENIC AVIAN INFLUENZA IN EUROPE

Between October 2020 and the summer of 2021, Europe experienced the largest and most severe avian influenza virus (AIV) epizootic ever in wild birds, poultry, and captive birds, caused by the emergence of clade 2.3.4.4b highly pathogenic AIV (HPAIV) of the H5Nx subtype. These heavy outbreaks resurged in October 2021 and continued in 2022. H5N8 HPAIVs initially dominated the epizootic until the spring of 2021. However, these viruses also reassorted with low-pathogenicity AIV (LPAIV), generating a wide range of genotypes in which internal genetic segments were swapped. In addition, reassortants featuring different neuraminidase (NA) subtypes (N1, N3, N4, and N5) were sporadically detected, mainly in wild birds in northwestern European regions, with some (e.g., H5N3) being detected in only a few wild-bird species or regions (1). Historically, severe epizootics of highly pathogenic avian influenza (HPAI) across Europe during winter seasons have been followed by a period of a sharp decline in HPAIV detections during the summer months to an average of 7 (range, 0 to 16) wild-bird cases (data according to the EMPRES-i database [https://empres-i.apps.fao.org/]) (see Table S1 in the supplemental material), presumably through the presence of immunity to the circulating hemagglutinin (HA) following high viral prevalences during the previous epizootic (2–4). In addition, the wide dispersal of birds during the breeding period provides unfavorable conditions for virus transmission during summer. However, since February 2021, the frequency of detection of the HPAIV H5N1 subtype has increased in late spring, and despite a decrease in cases throughout the summer, the virus did not completely disappear but was observed in 39 wild birds across Europe, challenging the restricted seasonal occurrence of HPAIV in Europe so far (1). While most poultry outbreaks detected until the summer of 2021 were caused by the H5N8 subtype, the H5N1 virus started to dominate in wild birds in the late spring of 2021, reversing the autumn/winter 2020–2021 scenario, during which H5N8 was the most widespread genotype in almost all of the affected species. The HPAIV H5N1 infections, sporadically detected throughout the summer of 2021 and in early autumn in wild birds, were all reported in northern Europe, in an area between the British Outer Hebrides and the Gulf of Finland (1, 5). In addition, cases also occurred along the Russia-Kazakhstan border. Coinciding with the onset of the autumn migration of aquatic wild birds, a renewed spike of HPAIV H5N1 detections in wild birds was observed, initially affecting apparently healthy Eurasian wigeon (Mareca penelope) and teal (Anas crecca) and later on being associated with increased deaths. The first detections were observed on the Wadden Sea coast of Germany and Denmark in October 2021. After a short delay, associated outbreaks were also detected in zoos and poultry farms in parallel with an increasing number of wild-bird cases across Europe (1).

10.1128/mbio.00609-22.4 TABLE S1 HPAIV cases in wild birds and outbreaks in poultry in Europe during summertime. Download Table S1, DOCX file, 0.01 MB. Copyright © 2022 Pohlmann et al. 2022 Pohlmann et al. https://creativecommons.org/licenses/by/4.0/ This content is distributed under the terms of the Creative Commons Attribution 4.0 International license.

The sequence of events described above raises several questions. Which reservoir species have enabled the maintenance and reemergence of HPAIV H5N1 causing the extensive epizootic in late 2021 and early 2022? Have the outbreaks toward the end of 2021 been caused by viruses that were maintained in Europe, and/or were these viruses reintroduced by migratory birds, and if so, did these viruses replicate/evolve in the European Union or at breeding sites in northeast Eurasia? Finally, why has previous exposure of wild birds during the H5N8 epizootic from 2020 to 2021 not led to a reduced incidence in the 2021–2022 autumn/winter season?

## PHYLOGENETIC ANALYSES REVEAL THE COCIRCULATION OF AT LEAST TWO HEMAGGLUTININ LINEAGES WITHIN CLADE 2.3.4.4b IN EUROPE IN 2021

**FIG 1:**
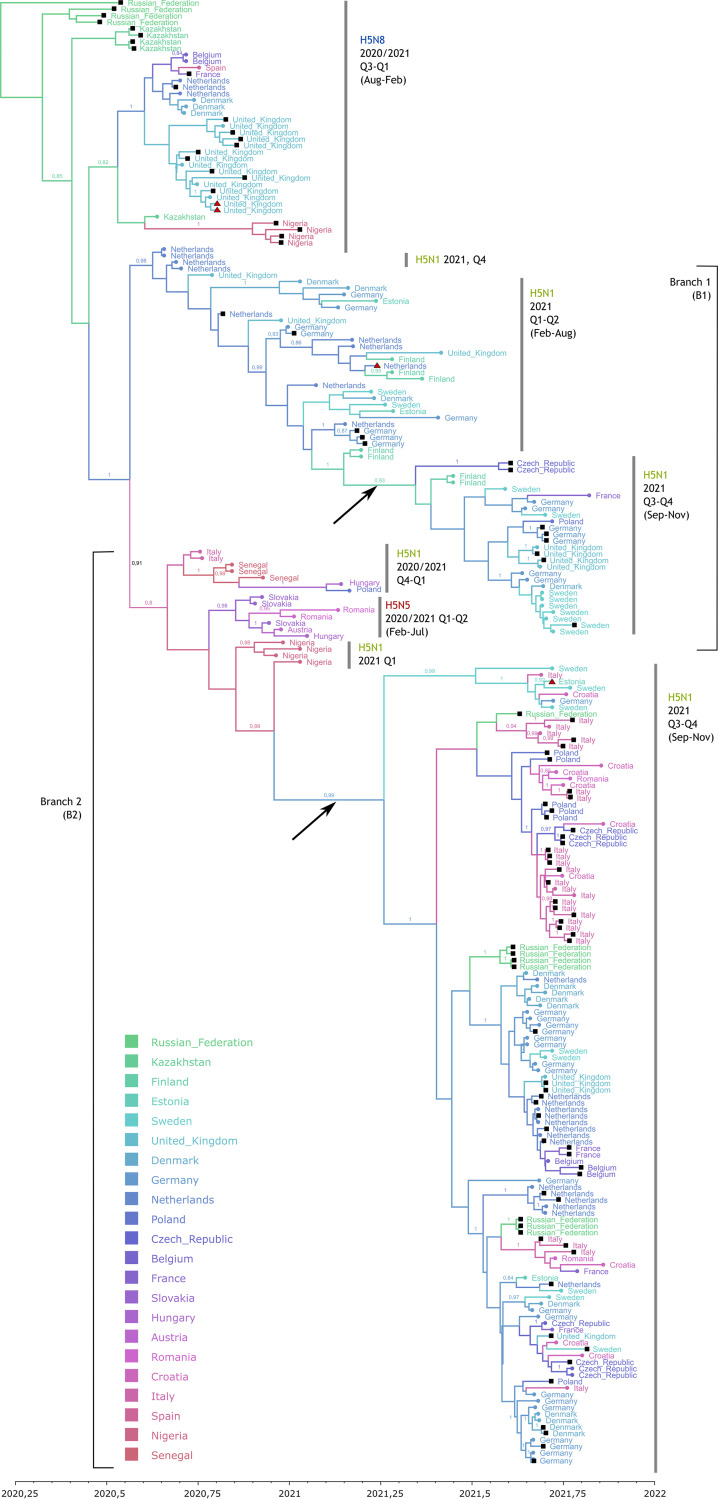
Time-scaled maximum clade credibility (MCC) phylogeny of hemagglutinin (HA) sequences of HPAIV collected between August 2020 and November 2021 (scale at the bottom) in wild birds (colored round tips), poultry (black square tips), and mammals (red triangle tips) of European countries. Country colors are ordered from northeast to southwest. Two distinct branches, B1 and B2, as well as the times and subtypes of different sublineages are depicted. The two arrows indicate a time gap during the summer period in branches B1 and B2.

**FIG 2:**
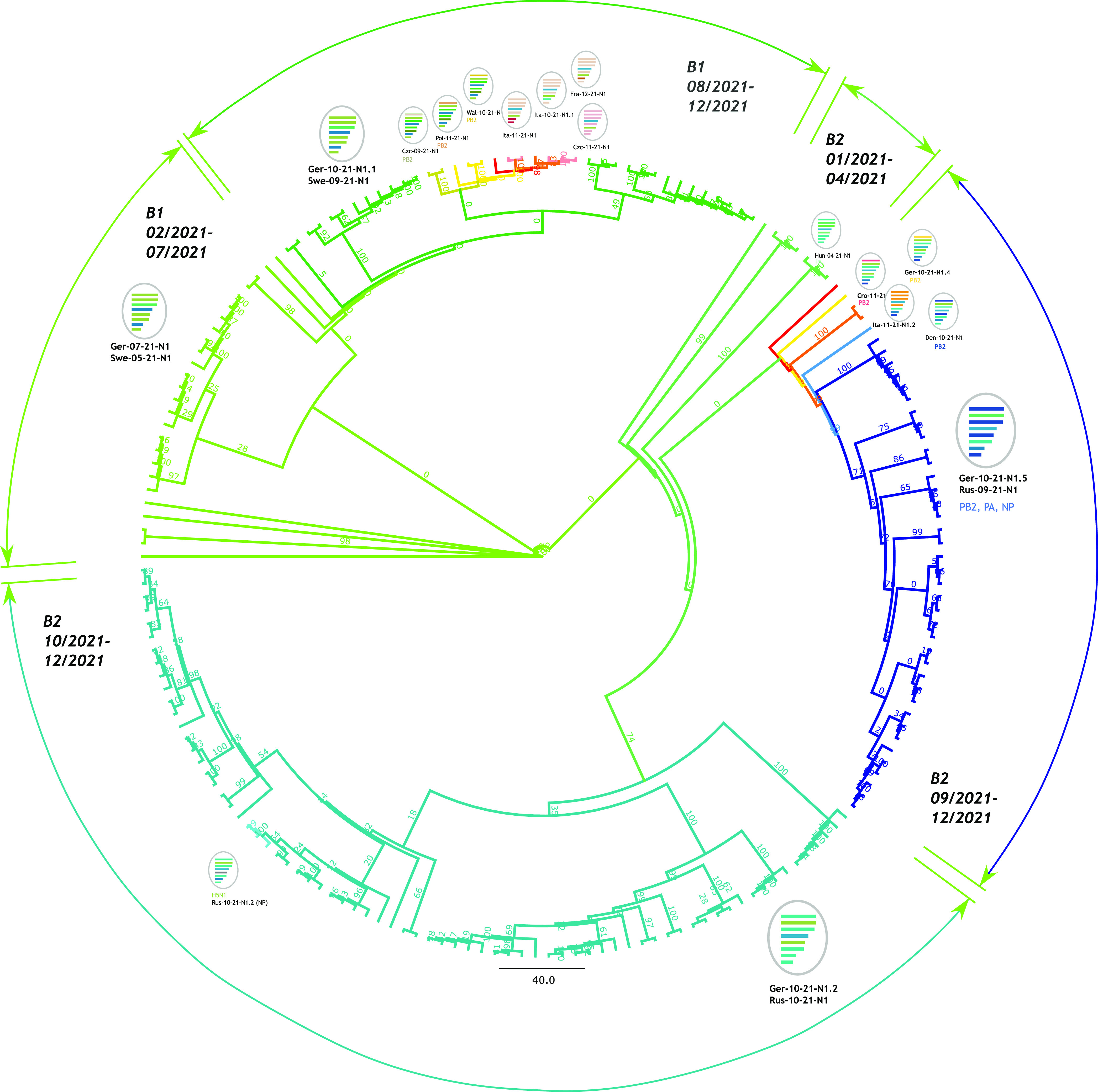
Genotype-based panoramic scope of reassortant HPAIV H5N1 detected in Europe in 2021. Maximum likelihood (ML) trees of concatenated coding sequences of full genomes were generated with RAxML. A polar tree layout with proportional transformed branches was used for visualization. The genome composition is sketched with appropriately colored segments for each genotype; major genotypes are also shown as schematic virions.

Currently available AIV sequence data established here and taken from public databases were compiled and analyzed phylogenetically (maximum likelihood [ML] and maximum credibility analyses). Of these sequences, 240 originated from viruses detected in wild birds, while 106 came from poultry (Table S2). Phylogenetic analyses of the H5 HA gene revealed that two H5 sublineages within clade 2.3.4.4b, tentatively termed B1 (“branch” 1) and B2, have cocirculated since the autumn of 2021 in Europe (Fig. 1; an identical tree with full sequence names and accession numbers is provided in Fig. S1). The divergence within these sublineages amounts to 0 to 9 nucleotides (B1 lineage) compared to 2 to 18 nucleotides (B2 lineage), which result in up to 3 (B1) and 5 (B2) amino acid differences. A lineage-indicating substitution is present at HA position 548 (HA548), coding for methionine in B1 and isoleucine in B2. The close phylogenetic relationship of the viruses of the B1 sublineage, which form a monophyletic branch in the Bayesian HA phylogeny; its stepwise branching pattern throughout the evolutionary period; and a consecutive temporal order support its continuous circulation at a relatively low level in northwestern Europe and Scandinavia during the summer of 2021. However, lineage B1 reveals a small time gap in the pectinate pattern (Fig. 1, arrow B1). A significantly larger time gap is evident in the second monophyletic lineage, B2, between the Nigerian poultry HPAIV sequences of March 2021 (e.g., EPI_ISL_4061491) and a virus detected in a jackdaw (EPI_ISL_7050532) in August 2021 in Sweden (Fig. 1, arrow B2). These “summer gaps,” which could not be closed by including contemporary sequences from locations outside Europe, likely indicate missing bridging sequences as AIVs from relevant species and/or geographic regions have been undersampled. Although the B1 and B2 branches are also supported by high bootstrap values in the corresponding maximum likelihood tree (Fig. S2), such summer gaps in genetic distances are not clearly visible. Thus, presumably, viruses have been maintained at a low level of circulation during the summer months of 2021 in Europe (B1) or other geographic areas (B2). The increasing numbers of H5N1 reassortants/genotypes, from a single event since February 2021 (Fig. 2, light green) to at least 16 events until November 2021 (Fig. 2, other colors), give further evidence supporting maintenance through sustained circulation. However, it cannot be excluded that both defined sublineages arose from two separate precursors within the range of H5 HPAIVs identified in Europe, Russia, and Africa since July 2020 (Fig. 1).

10.1128/mbio.00609-22.2 FIG S1 Time-scaled maximum clade credibility (MCC) phylogeny of hemagglutinin (HA) sequences of HPAIV collected between August 2020 and November 2021. Geographical and species origins can be retrieved from the list of viruses shown to the right of the tree. The tree is identical to that in Fig. 1, with the added details of full sequence names and accession numbers. Download FIG S1, EPS file, 0.3 MB. Copyright © 2022 Pohlmann et al. 2022 Pohlmann et al. https://creativecommons.org/licenses/by/4.0/ This content is distributed under the terms of the Creative Commons Attribution 4.0 International license.

10.1128/mbio.00609-22.3 FIG S2 Maximum likelihood phylogeny of hemagglutinin sequences of HPAIV collected between August 2020 and November 2021 in wild birds, poultry, and mammals of European countries. Download FIG S2, EPS file, 0.1 MB. Copyright © 2022 Pohlmann et al. 2022 Pohlmann et al. https://creativecommons.org/licenses/by/4.0/ This content is distributed under the terms of the Creative Commons Attribution 4.0 International license.

10.1128/mbio.00609-22.5 TABLE S2 Overview of sequence metadata. Download Table S2, DOCX file, 0.04 MB. Copyright © 2022 Pohlmann et al. 2022 Pohlmann et al. https://creativecommons.org/licenses/by/4.0/ This content is distributed under the terms of the Creative Commons Attribution 4.0 International license.

The B1 HPAI H5N1 viruses, first identified in wigeons in the Wadden Sea in week 41 in 2021, are highly similar to the HPAI H5N1 viruses detected for the first time in the fourth quarter (Q4) of 2020 in, e.g., The Netherlands (for example, the genotypes NEL 2020 and GER 2020-02 showed 99.8% identity, and the identity to GER Q3Q4 is 99.6% across the coding regions of all segments). This strain was present in Germany in Q1 and Q2 of 2021 as well as in other northern European countries in 2021. Full-genome sequence analyses revealed that these viruses represent a single genotype continuum (Ger-02-21-N1, Ger-10-21-1.1, and Ger-10-21-1.3) (Fig. 1 and 2).

However, after week 42, an expansion became evident in the spectrum of genotypes of the H5N1 HPAIVs in Europe, showing reassortment of the PB2, PA, and NP segments of a currently unknown origin, i.e., in LPAIVs circulating in wild birds in Asia or Europe (genotypes Ger-10-21-N1.4 and -1.5). These genotypes were associated with an H5 HA of B2, which was also found in the genotype background of Ger-10-21-N1.2 (Fig. 2).

## EVIDENCE FOR LOCAL CIRCULATION AND INDEPENDENT NEW INCURSIONS OF SUBLINEAGES B1 AND B2, RESPECTIVELY

From the data available, these intricate relationships indicate that at least one further independent incursion into northern Europe of an HPAI H5N1 virus with an HA of sublineage B2 must have occurred in October 2021. It is important to note that H5 sequences from Senegal and Nigeria (February to March 2021) are in the younger ancestry of this lineage, although its eldest ancestors were detected in Italy in November 2020. Closely related B1 viruses circulated during the winter of 2020 in southeast Europe (as reassortants with an N5 NA partner), and viruses of this lineage were introduced from Europe to western Africa in the winter of 2020 (6), affecting Nigerian poultry in March 2021 (Fig. 1). Unfortunately, no sequences are available from these regions during the summer period, but it could be speculated that the summer gap was associated with northeastward wild-bird spring migration, transposing the virus from Africa toward the Near East and Central Asia. Strikingly, HA sequences from HPAI H5N1 outbreaks on poultry farms in southern Russia in the autumn of 2021 cluster with B2 HA genes (Fig. 1). However, the analyses of the full genomes reveal evidence for different genotypes with reassorted genetic segments that have emerged from geographically disparate poultry farms across Russia. Likewise, other HPAI H5N1 viruses detected in poultry in central Europe during Q3 of 2021 (e.g., in the Czech Republic) present a different genotype with a reassorted PB2 gene segment (Czc-09-21-N1) (Fig. 2). The emergence of multiple regionally restricted genotypes (e.g., Germany, Italy, Wales, Denmark, and Russia) underlines the unprecedented genomic variety of the circulating H5N1 viruses. The ancestral H5N1 virus in the winter of 2020 arose as a reassortant with PB2, PB1, PA, and NP segments from LPAIV collected in central Asia and NA1 and NS segments from European migratory wild-bird LPAIV. The ancestral strain for B1 and B2 further reassorted, incorporating PA from northern Asia and NP segments from central and northern Asia.

It likely requires additional sequences filling the indicated summer gap to clarify whether a set of HA B2 viruses of different genotypes (Ger-10-21-N1.2, -1.4, and -1.5) has been introduced or whether reassortment of PB2, PB1, PA, NP, and NS segments has occurred in northern or eastern Europe and to what extent poultry holdings play a role in the emergence of newly reassorted viruses. In addition, the lack of genomic information on LPAIV cocirculating in wild-bird populations both within Europe and across ecologically linked populations in Eurasia severely limits the accurate source designation of reassorted segments.

This study provides evidence for two temporally and geographically overlapping scenarios:

1. The continuous presence and genetic drift of HPAI H5N1 viruses related to genotype Ger-02-21-N1 and HA of the B1 type have been detected since the autumn of 2020 in northern Europe: representatives of this genotype persisted in wild birds in a localized discrete manner over the summer of 2021 in a wide geographic area stretching from the Outer Hebrides to the Bay of Finland, representing summer breeding grounds of those species, and touching the southern coasts of the North Sea and the Baltic Sea.
2. New substantive incursions of a second sublineage of HP H5N1 viruses (B2) have been detected since October 2021. This lineage showed an African ancestral relationship, in their HA genes, but was also detected contemporaneously in a wider area, including poultry outbreaks in Russia in September 2021. These viruses were initially (week 41 of 2021) detected in apparently healthy long-distance migratory birds (Eurasian wigeon and teal) in the Wadden Sea area.

## CONSEQUENCES OF THE EXPECTED INCREASE AND EXTENT OF HPAIV INFECTION PRESSURE ON WILD BIRDS AND POULTRY IN EUROPE

HPAIV H5N1 has emerged through uncertain pathways and drivers as the dominant HPAIV subtype (over previous H5N8 viruses) that is causing extensive infections across Europe since late 2021 and has even spread across the Atlantic to Newfoundland and extended its range into the United States (7, 8). The pattern of the rapid spread of the B1 and B2 sublineages of HPAIV H5N1 in migratory wild birds despite the preceding major epizootic affecting the same metapopulations of wild birds just half a year earlier has not been observed in Europe previously but may be due to unknown fitness advantages of the B1 and B2 HPAIVs (9). Although a high proportion of juvenile and highly susceptible migratory birds is expected in the autumn migratory period, at least some of the surviving adult wild birds should have been exposed to antigenically similar H5 molecules during the previous season and, as such, would be predicted to have some level of preexposure immunity (10–12). Currently, it is not clear whether juvenile wild birds are preferentially affected by current HPAIV H5N1 infections; likewise, there is no indication that there have been significant die-offs in some hosts such as dabbling ducks. Clearly, highly pathogenic H5N1 is altering both its host range and epidemiology within wild-bird hosts. Detailed future characterization of the viruses prevalent in early and late 2021 should include antigenic cartography with both birds and ferret antisera to investigate whether, apart from proven genetic drift, some antigenic drift contributed to the selection and success of these sublineages. However, an *in silico* analysis of current H5N1 and previous H5N8-related HA sequences according to Peeters et al. (13) did not suggest amino acid substitutions at sites that are critical for antigenicity (data not shown).

The available data suggest a fundamental shift in the observed epidemiology of these viruses, with the potential for an enzootic status of this HPAIV in northern Europe (“enzootic scenario”), together with repeated incursion risks via migratory birds. This observation has highly significant implications for both prevention and control strategies as extended (annual instead of seasonal) and increased (by numbers of infected wild birds per month) incursion pressure ensues for European poultry holdings. However, the assessment of data needs to consider that our observations are based on no more than 12 months of evidence, with a surveillance gap being strongly acknowledged in our assessment. Further to recurrent epizootics, the repeated detection of influenza of avian origin in wild mammals (Estonia, Finland, Germany, Sweden, The Netherlands, and the United Kingdom [14]) emphasizes the need to characterize the zoonotic potential of these emerging genotypes. Optimized biosecurity measures, early detection, rapid containment, and depopulation of the affected premises remain the backbone of defense against incursions in areas where vaccination is not practiced (15). However, infection pressure is expected to remain high, and it is extended temporally. High vigilance and maintaining effective biosecurity measures are strenuous for poultry holders. There are also some conflicts of goals affecting biosecurity; i.e., forced housing of free-range poultry has to be limited for both animal welfare and economic/marketing reasons. Certainly, even with increased vigilance, future breaches in biosecurity defenses are likely (16).

In conclusion, we propose that further preventative tools must be considered to protect the poultry production sector in vulnerable regions (wetland areas with dense poultry populations) and in critical poultry sectors (turkey fattening and free-range table egg production). Since proximity to open water bodies has been identified as a risk factor, avoiding the placement of poultry farms close to wetland areas as well as carefully planned and controlled vaccinations of flocks at risk should be considered (17–20). However, both options require a fundamental shift in the approach to preventive measures, especially across countries that currently favor surveillance and culling over vaccination. Further investment in next-generation vaccines that can be available at low cost and well matched to viruses circulating contemporaneously is required while supporting global approaches for improved control and threat mitigation.

10.1128/mbio.00609-22.1 TEXT S1 Technical details related to sample origin, sequencing methodology, and phylogenetic analyses, including specific references. Download Text S1, DOCX file, 0.02 MB. Copyright © 2022 Pohlmann et al. 2022 Pohlmann et al. https://creativecommons.org/licenses/by/4.0/ This content is distributed under the terms of the Creative Commons Attribution 4.0 International license.
