# Supplementary material for: Has Epizootic Become Enzootic? Evidence for a Fundamental Change in the Infection Dynamics of Highly Pathogenic Avian Influenza in Europe, 2021
Source: mBio. 2022 Jun 21;13(4):e00609-22. doi: 10.1128/mbio.00609-22 (PMC9426456; doi:10.1128/mbio.00609-22)
Supplement: TABLE S1 [file mbio.00609-22-s0001.docx]

| **Year^1^** | **Wild birds cases** | **Poultry outbreaks** |
| --- | --- | --- |
| 2006 | 6 | 16 |
| 2007 | 16 | 5 |
| 2017 | 8 | 35 |
| 2018 | 5 | 4 (>50 western Russia) |
| 2020 | 0 | 4 |
| 2021 | **39** | 12 |

**Supplemental table 1.** HPAIV cases in wild birds and outbreaks in poultry in Europe during summer time.

^1^ – Summer time period: 01.06 – 31.08.

Data according to the EMPRESS-I database at https://empres-i.apps.fao.org/
