# Supplementary material for: Has Epizootic Become Enzootic? Evidence for a Fundamental Change in the Infection Dynamics of Highly Pathogenic Avian Influenza in Europe, 2021
Source: mBio. 2022 Jun 21;13(4):e00609-22. doi: 10.1128/mbio.00609-22 (PMC9426456; doi:10.1128/mbio.00609-22)
Supplement: TABLE S2 [file mbio.00609-22-s0002.docx]

**Supplementary table 2.** Overview of sequence metadata.

| **Virus designation** | **Subtype** | **Lineage** | **Collection date** | **Host species** | **Country** | **Wild** | **Accession** |
| --- | --- | --- | --- | --- | --- | --- | --- |
| A/Eurasian_Wigeon/Netherlands/1/2020 | H5N1 | B1 | 2020-10-16 | eurasian wigeon | Netherlands | Y | 603133 |
| A/greylag_goose/Netherlands/20016582-004/2020 | H5N1 | B1 | 2020-10-28 | greylag goose | Netherlands | y | 632314 |
| A/pheasant/Scotland/000348/2021 | H5N1 | B1 | 2021-02-10 | pheasant | United Kingdom | y | 1123361 |
| A/Eurasian_Wigeon/Netherlands/4/2020 | H5N1 | B1 | 2020-10-16 | eurasian wigeon | Netherlands | y | 603134 |
| A/eurasian teal/Netherlands/20016896-013/2020 | H5N1 | B1 | 2020-11-02 | eurasian teal | Netherlands | y | 632315 |
| A/chicken/Netherlands/20019879-001005/2020 | H5N1 | B1 | 2020-12-14 | chicken | Netherlands | n | 711055 |
| A/barnacle goose/Germany-NI/AI03980/2021 | H5N1 | B1 | 2021-04-14 | barnacle goose | Germany | y | 5146291 |
| A/barnacle goose/Germany-NI/AI03914/2021 | H5N1 | B1 | 2021-04-08 | barnacle goose | Germany | y | 5145422 |
| A/barnacle_goose/Denmark/17572-1.01/2021-03-01 | H5N1 | B1 | 2021-03-01 | barnacle goose | Denmark | y | 5449305 |
| A/mute_swan/Austria/21013162_21VIR1085-5/2021 | H5N5 |  | 2021-02-10 | mute swan | Austria | y | 1665258 |
| A/mute_swan/Slovakia/Pah15_21VIR1086-3/2021 | H5N5 |  | 2021-01-29 | mute swan | Slovakia | y | 1665261 |
| A/eagle/Hungary/8569/2021 | H5N5 |  | 2021-03-08 | eagle | Hungary | y | 3135926 |
| A/mute_swan/Slovakia/Pah6_21VIR1086-2/2021 | H5N5 |  | 2021-01-15 | mute swan | Slovakia | y | 1665262 |
| A/Eurasian_wigeon/Italy/20VIR7301-206/2020 | H5N1 |  | 2020-11-21 | eurasian wigeon | Italy | y | 683592 |
| A/chicken/Romania/10101_21VIR2044-1/2021 | H5N5 |  | 2021-02-23 | chicken | Romania | n | 1665249 |
| A/chicken/Slovakia/Pah10_21VIR1086-5/2021 | H5N5 |  | 2021-01-22 | chicken | Slovakia | n | 1665250 |
| A/greater_white-fronted_goose/Italy/20VIR8073-4/2020 | H5N1 |  | 2020-11-23 | greater white-fronted goose | Italy | y | 956412 |
| A/chicken/Senegal/21VIR1084-4/2021 | H5N1 |  | 2020-12-23 | chicken | Senegal | n | 2276069 |
| A/chicken/Senegal/21VIR1084-3/2021 | H5N1 |  | 2020-12-23 | chicken | Senegal | n | 2276068 |
| A/common_murre/Netherlands/21025491-002/2021 | H5N1 | B1 | 2021-03-17 | common murre | Netherlands | y | 2174084 |
| A/oystercatcher/Germany-NI/AI05047/2021 | H5N1 | B1 | 2021-07-17 | oystercatcher | Germany | y | 5145744 |
| A/chicken/Germany-NI/AI01599/2021 | H5N1 | B1 | 2021-02-23 | chicken | Germany | n | 5099451 |
| A/barnacle goose/Germany-NI/AI01605/2021 | H5N1 | B1 | 2021-02-17 | barnacle goose | Germany | y | 5099456 |
| A/barnacle_goose/Netherlands/21028196-002/2021 | H5N1 | B1 | 2021-04-22 | barnacle goose | Netherlands | y | 2194036 |
| A/pheasant/Wales/385129/2021 | H5N1 | B1 | 2021-10-27 | pheasant | United Kingdom | y | 9012572 |
| A/chicken/Wales/053969/2021 | H5N1 | B1 | 2021-10-30 | chicken | United Kingdom | n | 9012618 |
| A/barnacle_goose/Finland/6378_21VIR7689-7/2021 | H5N1 | B1 | 2021-05-01 | barnacle goose | Finland | y | 7778774 |
| A/European_herring_gull/Finland/9722_21VIR7689-13/2021 | H5N1 | B1 | 2021-08-01 | european herring gull | Finland | y | 7778766 |
| A/Eurasian_eagle-owl/Finland/10617_21VIR7689-15/2021 | H5N1 | B1 | 2021-08-01 | eurasian eagle-owl | Finland | y | 7778768 |
| A/barnacle_goose/Finland/6247_21VIR7689-6/2021 | H5N1 | B1 | 2021-05-01 | barnacle goose | Finland | y | 7778773 |
| A/domestic duck/Germany-BB/AI06239/2021 | H5N1 | B1 | 2021-11-02 | domestic duck | Germany | n | 11725869 |
| A/chicken/Germany-BB/AI06219/2021 | H5N1 | B1 | 2021-10-29 | chicken | Germany | n | 11725962 |
| A/black-headed gull/Germany-NI/AI05962/2021 | H5N1 | B1 | 2021-10-12 | black-headed gull | Germany | y | 7733048 |
| A/pelican/France/21P013720/2021 | H5N1 | B1 | 2021-12-15 | pelican | France | y | 8377056 |
| A/chicken/Germany-BB/AI06242/2021 | H5N1 | B1 | 2021-11-02 | chicken | Germany | n | 11725988 |
| A/chicken/England/053052/2021 | H5N1 | B1 | 2021-10-24 | chicken | United Kingdom | n | 9012457 |
| A/mute_swan/England/053054/2021 | H5N1 | B1 | 2021-10-24 | mute swan | United Kingdom | y | 5804708 |
| A/duck/Czech_Republic/18520-2/2021 | H5N1 | B1 | 2021-09-27 | duck | Czech Republic | n | 5323347 |
| A/goose/Czech_Republic/18520-1/2021 | H5N1 | B1 | 2021-09-27 | goose | Czech Republic | n | 5323346 |
| A/duck/Kazakhstan/12-20-B-Talg-11/2020 | H5N8 |  | 2020-09-15 | duck | Kazakhstan | n | 2932609 |
| A/pigeon/Kazakhstan/15-20-B-Talg-5/2020 | H5N8 |  | 2020-09-15 | pigeon | Kazakhstan | y | 2932682 |
| A/goose/Kazakhstan/7-20-B-Talg-12/2020 | H5N8 |  | 2020-09-22 | goose | Kazakhstan | n | 2932612 |
| A/crow/Kazakhstan/15-20-B-Talg-4/2020 | H5N8 |  | 2020-09-15 | crow | Kazakhstan | y | 2932617 |
| A/chicken/Kazakhstan/1-20-B-Talg-67/2020 | H5N8 |  | 2020-10-09 | chicken | Kazakhstan | n | 2932683 |
| A/chicken/Nigeria/VRD21-43_21VIR2288-4/2021 | H5N8 |  | 2021-02-10 | chicken | Nigeria | n | 4061481 |
| A/chicken/Nigeria/VRD21-100_21VIR2370-423/2021 | H5N8 |  | 2021-03-01 | chicken | Nigeria | n | 4061483 |
| A/chicken/Nigeria/VRD21-035B_21VIR2288-1/2021 | H5N8 |  | 2021-02-05 | chicken | Nigeria | n | 4061482 |
| A/chicken/Nigeria/VRD21-53B_21VIR2288-5/2021 | H5N8 |  | 2021-02-11 | chicken | Nigeria | n | 4061488 |
| A/great-white_pelican/Senegal/21-67_21VIR1084-8/2021 | H5N1 |  | 2021-01-23 | great-white pelican | Senegal | y | 2276071 |
| A/white_stork/Poland/MB391/2021 | H5N1 |  | 2021-04-20 | white stork | Poland | y | 2681045 |
| A/Turkey/Hungary/16603/2021 | H5N1 |  | 2021-04-13 | turkey | Hungary | n | 3135897 |
| A/barnacle_goose/Denmark/19027-1.02/2021 | H5N1 | B1 | 2021-04-18 | barnacle goose | Denmark | y | 11798566 |
| A/white-tailed_eagle/Estonia/TA2111864-2_21VIR7512-6/2021 | H5N1 | B1 | 2021-05-16 | white-tailed eagle | Estonia | y | 7778755 |
| A/chicken/Nigeria/VRD21-37_21VIR2288-2/2021 | H5N1 |  | 2021-02-05 | chicken | Nigeria | n | 4061486 |
| A/herring gull/Germany-SH/AI06141/2021 | H5N1 | B1 | 2021-10-21 | herring gull | Germany | y | 7753347 |
| A/barnacle_goose/Finland/6955_21VIR7689-9/2021 | H5N1 | B1 | 2021-06-01 | barnacle goose | Finland | y | 7778775 |
| A/white-tailed_eagle/Netherlands/21027616-001/2021 | H5N1 | B1 | 2021-04-23 | white-tailed eagle | Netherlands | y | 2194014 |
| A/golden_eagle/Finland/9378_21VIR7689-12/2021 | H5N1 | B1 | 2021-07-01 | golden eagle | Finland | y | 7778765 |
| A/white-tailed_eagle/Finland/6984_21VIR7689-10/2021 | H5N1 | B1 | 2021-06-01 | white-tailed eagle | Finland | y | 7778763 |
| A/fox/Netherlands/21028774-002/2021 | H5N1 | B1 | 2021-05-17 | fox | Netherlands | y | 2194218 |
| A/gull/Estonia/TA2113284-4_21VIR7512-8/2021 | H5N1 | B1 | 2021-06-02 | gull | Estonia | y | 7778756 |
| A/turkey/Germany-NI/AI04455/2021 | H5N1 | B1 | 2021-05-05 | turkey | Germany | n | 5145223 |
| A/turkey/Germany-NI/AI04373/2021 | H5N1 | B1 | 2021-04-27 | turkey | Germany | n | 5146292 |
| A/barnacle_goose/Netherlands/21027016-002/2021 | H5N1 | B1 | 2021-04-15 | barnacle goose | Netherlands | y | 2193998 |
| A/turkey/Germany-NI/AI04425/2021 | H5N1 | B1 | 2021-05-03 | turkey | Germany | n | 5146481 |
| A/European_herring_gull/Denmark/19968-1.02/2021 | H5N1 | B1 | 2021-05-14 | european herring gull | Denmark | y | 11798575 |
| A/mute_swan/Poland/MB490-L1/2021 | H5N1 | B1 | 2021-11-08 | mute swan | Poland | y | 6937114 |
| A/Eurasian wigeon/Germany-SH/AI05948/2021 | H5N1 | B1 | 2021-10-09 | eurasian wigeon | Germany | y | 5403566 |
| A/Eurasian curlew/Germany-SH/AI05960/2021 | H5N1 | B1 | 2021-10-14 | eurasian curlew | Germany | y | 7748001 |
| A/common_buzzard_/Sweden/SVA211104SZ0320/FB004419/M-2021 | H5N1 | B1 | 2021-10-29 | common buzzard | Sweden | y | 6600769 |
| A/barnacle_goose/Sweden/SVA211102SZ0402/FB004395/M-2021 | H5N1 | B1 | 2021-11-02 | barnacle goose | Sweden | y | 6596216 |
| A/common_pheasant_/Sweden/SVA211104SZ0320/FB004417/M-2021 | H5N1 | B1 | 2021-10-29 | common pheasant | Sweden | y | 6599895 |
| A/barnacle_goose/Denmark/24342-1.02/2021 | H5N1 | B1 | 2021-10-30 | barnacle goose | Denmark | y | 11798569 |
| A/great_black-backed_gull/Sweden/SVA211109SZ0434/FB004445/M-2021 | H5N1 | B1 | 2021-11-09 | great black-backed gull | Sweden | y | 6600943 |
| A/common_buzzard/Sweden/SVA211111SZ0376/FB004484/2021 | H5N1 | B1 | 2021-11-09 | common buzzard | Sweden | y | 7053000 |
| A/common_pheasant_/Sweden/SVA211104SZ0320/FB004418/M-2021 | H5N1 | B1 | 2021-10-29 | common pheasant | Sweden | y | 6600745 |
| A/Chicken/Sweden/SVA211130SZ0427/FB290424-IP-1/M-2021 | H5N1 | B1 | 2021-11-30 | chicken | Sweden | n | 7452805 |
| A/mute_swan/Romania/11981-1_21VIR3163-5/2021 | H5N5 |  | 2021-04-08 | mute swan | Romania | y | 3102081 |
| A/chicken/Nigeria/VRD21-109_21VIR2370-425/2021 | H5N1 |  | 2021-03-01 | chicken | Nigeria | n | 4061485 |
| A/western_jackdaw/Sweden/SVA211111SZ0376/FB004483/2021 | H5N1 | B2 | 2021-11-08 | western jackdaw | Sweden | y | 7050532 |
| A/greylag_goose_/Sweden/SVA211103SZ0398/FB004410/2021 | H5N1 | B1 | 2021-11-01 | greylag goose | Sweden | y | 6599075 |
| A/greylag_goose/Netherlands/20016879-001/2020 | H5N8 |  | 2020-11-01 | greylag goose | Netherlands | y | 632318 |
| A/chicken/England/033708/2020 | H5N8 |  | 2020-11-09 | chicken | United Kingdom | n | 710509 |
| A/barnacle_goose/Denmark/14600-2/2020 | H5N8 |  | 2020-11-07 | barnacle goose | Denmark | y | 984692 |
| A/chicken/Netherlands/20016597-026030/2020 | H5N8 |  | 2020-10-28 | chicken | Netherlands | n | 603132 |
| A/barnacle_goose/Netherlands/20016935-002/2020 | H5N8 |  | 2020-11-01 | barnacle goose | Netherlands | y | 632317 |
| A/whistling_duck/England/035643/2020 | H5N8 |  | 2020-11-18 | whistling duck | United Kingdom | y | 710512 |
| A/chicken/Northern_Ireland/2020-17671_21VIR113-11/2020 | H5N8 |  | 2020-12-31 | chicken | United Kingdom | n | 995172 |
| A/Numenius_arquata/Belgium/11956_003/2020 | H5N8 |  | 2020-11-07 | eurasian curlew | Belgium | y | 664102 |
| A/Anser_albifrons/Belgium/11956_005/2020 | H5N8 |  | 2020-11-07 | greater white-fronted goose | Belgium | y | 661313 |
| A/Canada_goose/England/032697/2020 | H5N8 |  | 2020-11-03 | canada goose | United Kingdom | y | 710506 |
| A/peregrine_falcon/Spain/3365-1_21VIR1230-1/2020 | H5N8 |  | 2020-11-20 | peregrine falcon | Spain | y | 2234814 |
| A/falcon/England/041976/2020 | H5N8 |  | 2020-12-14 | falcon | United Kingdom | y | 766056 |
| A/barnacle_goose/Denmark/14536-1/2020 | H5N8 |  | 2020-11-05 | barnacle goose | Denmark | y | 984686 |
| A/chicken/Northern_Ireland/2021-000067_21VIR114-19/2021 | H5N8 |  | 2021-01-05 | chicken | United Kingdom | n | 996003 |
| A/duck/England/043628/2020 | H5N8 |  | 2020-12-17 | duck | United Kingdom | n | 1123351 |
| A/chicken/England/045984/2020 | H5N8 |  | 2020-12-24 | chicken | United Kingdom | n | 1123352 |
| A/turkey/England/038730/2020 | H5N8 |  | 2020-12-03 | turkey | United Kingdom | n | 766052 |
| A/chicken/France/20P016448/2020 | H5N8 |  | 2020-11-10 | chicken | France | n | 667810 |
| A/chicken/England/046491/2020 | H5N8 |  | 2020-12-28 | chicken | United Kingdom | n | 1123354 |
| A/barnacle_goose/Denmark/14538-1/2020 | H5N8 |  | 2020-11-15 | barnacle goose | Denmark | y | 984688 |
| A/seal/England/AVP-031141/2020 | H5N8 |  | 2020-12-08 | seal | United Kingdom | y | 2081528 |
| A/turkey/England/039352/2020 | H5N8 |  | 2020-12-04 | turkey | United Kingdom | n | 766053 |
| A/mute_swan/England/234135/2020 | H5N8 |  | 2020-12-01 | mute swan | United Kingdom | y | 1123360 |
| A/chicken/England/037052/2020 | H5N8 |  | 2020-11-19 | chicken | United Kingdom | n | 710511 |
| A/red_fox/England/AVP-M1-21-01/2020 | H5N8 |  | 2020-12-08 | red fox | United Kingdom | y | 2081527 |
| A/common_pheasant_/Sweden/SVA210923SZ0341/KN000366/M-2021 | H5N1 | B1 | 2021-09-22 | common pheasant | Sweden | y | 4653177 |
| A/chicken/Nigeria/VRD21-98_21VIR2288-6/2021 | H5N1 |  | 2021-02-12 | chicken | Nigeria | n | 4061491 |
| A/barnacle_goose/Sweden/SVA210511SZ0567/FB001840/M-2021 | H5N1 | B1 | 2021-05-11 | barnacle goose | Sweden | y | 3005950 |
| A/chicken/Nigeria/VRD21-102_21VIR2370-424/2021 | H5N1 |  | 2021-03-01 | chicken | Nigeria | n | 4061484 |
| A/White-Tailed_Eagle/Sweden/SVA210528SZ0223/KN002027/AB-2021 | H5N1 | B1 | 2021-05-25 | white-tailed eagle | Sweden | y | 2652003 |
| A/mute_swan/England/234255/2020 | H5N1 | B1 | 2020-12-03 | mute swan | United Kingdom | y | 766876 |
| A/great_skua/Scotland/041672/2021 | H5N1 | B1 | 2021-07-20 | great skua | United Kingdom | y | 5530613 |
| A/chicken/Kurgan/1001/2020 | H5N8 |  | 2020-08-27 | chicken | Russian Federation | n | 654834 |
| A/turkey/Omsk/0001/2020 | H5N8 |  | 2020-08-13 | turkey | Russian Federation | n | 644121 |
| A/goose/Omsk/30006/2020 | H5N8 |  | 2020-09-03 | goose | Russian Federation | n | 654829 |
| A/duck/Russian_Federation/Omsk/1328-2/2020 | H5N8 |  | 2020-08-17 | duck | Russian Federation | n | 626650 |
| A/mute_swan/Croatia/104/2021 | H5N1 | B2 | 2021-11-19 | mute swan | Croatia | y | 7570462 |
| A/turkey/Italy/21VIR10251/2021 | H5N1 | B2 | 2021-11-25 | turkey | Italy | n | 7733628 |
| A/chicken/Italy/21VIR10352/2021 | H5N1 | B2 | 2021-11-26 | chicken | Italy | n | 7733641 |
| A/chicken/Poland/H1940-N/2021 | H5N1 | B2 | 2021-11-05 | chicken | Poland | n | 6931288 |
| A/mute_swan/Croatia/144/2021 | H5N1 | B2 | 2021-12-27 | mute swan | Croatia | y | 8338456 |
| A/turkey/Italy/21VIR9618-7/2021 | H5N1 | B2 | 2021-11-18 | turkey | Italy | n | 7733587 |
| A/chicken/Italy/21VIR10388/2021 | H5N1 | B2 | 2021-11-30 | chicken | Italy | n | 7733582 |
| A/mallard/Italy/21VIR8919-2/2021 | H5N1 | B2 | 2021-10-27 | mallard | Italy | y | 7733591 |
| A/chicken/Italy/21VIR10384/2021 | H5N1 | B2 | 2021-11-30 | chicken | Italy | n | 7733607 |
| A/turkey/Italy/21VIR9510-1/2021 | H5N1 | B2 | 2021-11-11 | turkey | Italy | n | 7733635 |
| A/turkey/Italy/21VIR9512-1/2021 | H5N1 | B2 | 2021-11-11 | turkey | Italy | n | 7733632 |
| A/turkey/Italy/21VIR9649-2/2021 | H5N1 | B2 | 2021-11-15 | turkey | Italy | n | 7733643 |
| A/kestrel/Italy/21VIR10468/2021 | H5N1 | B2 | 2021-11-30 | kestrel | Italy | y | 7733642 |
| A/seagull/Italy/21VIR9432-2/2021 | H5N1 | B2 | 2021-11-11 | seagull | Italy | y | 7733621 |
| A/turkey/Poland/H1910-T3/2021 | H5N1 | B2 | 2021-11-01 | turkey | Poland | n | 6929958 |
| A/chicken/Italy/21VIR9133-20/2021 | H5N1 | B2 | 2021-11-04 | chicken | Italy | n | 7733617 |
| A/turkey/Poland/H1913-T1/2021 | H5N1 | B2 | 2021-11-02 | turkey | Poland | n | 6930238 |
| A/turkey/Poland/H1944-N/2021 | H5N1 | B2 | 2021-11-08 | turkey | Poland | n | 6935584 |
| A/chicken/Italy/21VIR10239/2021 | H5N1 | B2 | 2021-11-25 | chicken | Italy | n | 7733595 |
| A/goose/Czech_Republic/22608-1/2021 | H5N1 | B2 | 2021-11-18 | goose | Czech Republic | n | 8515478 |
| A/goose/Czech_Republic/22608-2/2021 | H5N1 | B2 | 2021-11-18 | goose | Czech Republic | n | 8515479 |
| A/mute_swan/Croatia/145/2021 | H5N1 | B2 | 2021-12-29 | mute swan | Croatia | y | 8338502 |
| A/chicken/Czech_Republic/23589-3/2021 | H5N1 | B2 | 2021-11-29 | chicken | Czech Republic | n | 7626532 |
| A/duck/Saratov/29-04/2021 | H5N1 | B2 | 2021-09-30 | duck | Russian Federation | n | 5463799 |
| A/chicken/Saratov/29-06/2021 | H5N1 | B2 | 2021-09-30 | chicken | Russian Federation | n | 5463800 |
| A/duck/Saratov/29-08/2021 | H5N1 | B2 | 2021-09-30 | duck | Russian Federation | n | 5463802 |
| A/duck/Saratov/29-02/2021 | H5N1 | B2 | 2021-09-30 | duck | Russian Federation | n | 5463797 |
| A/turkey/Italy/21VIR9767-3/2021 | H5N1 | B2 | 2021-11-17 | turkey | Italy | n | 7733624 |
| A/turkey/Italy/21VIR9652-2/2021 | H5N1 | B2 | 2021-11-15 | turkey | Italy | n | 7733629 |
| A/turkey/Italy/21VIR10340/2021 | H5N1 | B2 | 2021-11-29 | turkey | Italy | n | 7733597 |
| A/turkey/Italy/21VIR9143-2/2021 | H5N1 | B2 | 2021-11-03 | turkey | Italy | n | 7733633 |
| A/turkey/Italy/21VIR9210-1/2021 | H5N1 | B2 | 2021-11-05 | turkey | Italy | n | 7733647 |
| A/turkey/Italy/21VIR9210-1/2021 | H5N1 | B2 | 2021-11-05 | turkey | Italy | n | 7733647 |
| A/mute_swan/Croatia/101/2021 | H5N1 | B2 | 2021-11-12 | mute swan | Croatia | y | 7357590 |
| A/mute_swan/Romania/16790_21VIR11355/2021 | H5N1 | B2 | 2021-11-26 | mute swan | Romania | y | 8440976 |
| A/chicken/Tyumen/27-31/2021 | H5N1 | B2 | 2021-10-06 | chicken | Russian Federation | n | 5463793 |
| A/buzzard/Germany-SH/AI06210/2021 | H5N1 | B2 | 2021-10-26 | buzzard | Germany | y | 7753443 |
| A/magpie/Italy/21VIR9487-2/2021 | H5N1 | B2 | 2021-11-05 | magpie | Italy | y | 7733612 |
| A/chicken/Italy/21VIR10343/2021 | H5N1 | B2 | 2021-11-28 | chicken | Italy | n | 7733610 |
| A/goose/France/21P013228/2021 | H5N1 | B2 | 2021-11-25 | goose | France | n | 8377418 |
| A/barnacle goose/Germany-MV/AI06175/2021 | H5N1 | B2 | 2021-10-26 | barnacle goose | Germany | y | 11725991 |
| A/barnacle_goose/Sweden/SVA211111SZ0376/FB004496/2021 | H5N1 | B2 | 2021-11-01 | barnacle goose | Sweden | y | 7053817 |
| A/European_herring_gull/Sweden/SVA211116SZ0432/FB004518/M-2021 | H5N1 | B2 | 2021-11-08 | european herring gull | Sweden | y | 7054770 |
| A/chicken/Scotland/054477/2021 | H5N1 | B2 | 2021-11-01 | chicken | United Kingdom | n | 9012696 |
| A/European_Herring_Gull/Netherlands/21037412-002/2021 | H5N1 | B2 | 2021-10-25 | european Herring gull | Netherlands | y | 5945412 |
| A/eurasian teal/Denmark/24115-2/2021 | H5N1 | B2 | 2021-10-16 | eurasian teal | Denmark | y | 11798573 |
| A/Eurasian wigeon/Germany-SH/AI05953/2021 | H5N1 | B2 | 2021-10-14 | eurasian wigeon | Germany | y | 7753152 |
| A/mallard/Germany-NI/AI06010/2021 | H5N1 | B2 | 2021-10-17 | mallard | Germany | y | 7753230 |
| A/guinea_fowl/Scotland/054471/2021 | H5N1 | B2 | 2021-11-01 | guinea fowl | United Kingdom | y | 9012694 |
| A/greylag_goose/Denmark/24309-1.01/2021 | H5N1 | B2 | 2021-10-27 | greylag goose | Denmark | y | 11798578 |
| A/Barnacle_goose/Netherlands/21037293-001/2021 | H5N1 | B2 | 2021-10-25 | barnacle goose | Netherlands | y | 6025676 |
| A/duck/Scotland/054469/2021 | H5N1 | B2 | 2021-11-01 | duck | United Kingdom | n | 9012700 |
| A/chicken/Netherlands/21037287-006010/2021 | H5N1 | B2 | 2021-10-25 | chicken | Netherlands | n | 5588100 |
| A/black-headed_gull/Netherlands/21037589-002/2021 | H5N1 | B2 | 2021-10-27 | black-headed gull | Netherlands | y | 6101868 |
| A/Eurasian wigeon/Germany-SH/AI06142/2021 | H5N1 | B2 | 2021-10-22 | eurasian wigeon | Germany | y | 7753405 |
| A/Branta_leucopsis/Belgium/14735_0001/2021 | H5N1 | B2 | 2021-11-04 | barnacle goose | Belgium | y | 6761101 |
| A/Eurasian wigeon/Germany-SH/AI05951/2021 | H5N1 | B2 | 2021-10-14 | eurasian wigeon | Germany | y | 7753193 |
| A/chicken/Netherlands/21037708-006010/2021 | H5N1 | B2 | 2021-10-30 | chicken | Netherlands | n | 5804698 |
| A/greylag goose/Germany-SH/AI06205/2021 | H5N1 | B2 | 2021-10-25 | greylag goose | Germany | y | 7753425 |
| A/Eurasian_wigeon/Denmark/24066-9/2021 | H5N1 | B2 | 2021-10-13 | eurasian wigeon | Denmark | y | 11798572 |
| A/chicken/France/21P013076/2021 | H5N1 | B2 | 2021-11-25 | chicken | France | n | 8377417 |
| A/Gallus_gallus/Belgium/15977/2021 | H5N1 | B2 | 2021-12-06 | chicken | Belgium | n | 7880696 |
| A/Mute_swan/Netherlands/21037283-002/2021 | H5N1 | B2 | 2021-10-24 | Mute swan | Netherlands | y | 5804788 |
| A/Eurasian wigeon/Germany-SH/AI05955/2021 | H5N1 | B2 | 2021-10-14 | eurasian wigeon | Germany | y | 7753130 |
| A/greylag_goose/Denmark/24343-1.02/2021 | H5N1 | B2 | 2021-11-01 | greylag goose | Denmark | y | 11798579 |
| A/Gallus_gallus/Belgium/16070_003/2021 | H5N1 | B2 | 2021-12-07 | chicken | Belgium | n | 7880689 |
| A/goose/Netherlands/21037720-001/2021 | H5N1 | B2 | 2021-10-28 | goose | Netherlands | n | 6101869 |
| A/Eurasian_wigeon/Denmark/24279-1/2021 | H5N1 | B2 | 2021-10-24 | eurasian wigeon | Denmark | y | 11798574 |
| A/domestic goose/Germany-SH/AI06147/2021 | H5N1 | B2 | 2021-10-22 | domestic goose | Germany | n | 7753396 |
| A/chicken/Netherlands/21037907-006010/2021 | H5N1 | B2 | 2021-11-02 | chicken | Netherlands | n | 6328022 |
| A/chicken/Netherlands/21037233-001/2021 | H5N1 | B2 | 2021-10-22 | chicken | Netherlands | n | 5588106 |
| A/Eurasian teal/Germany-BY/AI05977/2021 | H5N1 | B2 | 2021-10-21 | eurasian teal | Germany | y | 7753367 |
| A/turkey/England/055251/2021 | H5N1 | B2 | 2021-11-06 | turkey | United Kingdom | n | 8814146 |
| A/barnacle goose/Germany-SH/AI06005/2021 | H5N1 | B2 | 2021-10-19 | barnacle goose | Germany | y | 7753251 |
| A/greylag goose/Germany-SH/AI06144/2021 | H5N1 | B2 | 2021-10-25 | greylag goose | Germany | y | 7753406 |
| A/chicken/Netherlands/21038165-006010/2021 | H5N1 | B2 | 2021-11-07 | chicken | Netherlands | n | 6101848 |
| A/Withe-tiled_eagle/Estonia/TA2124126-1_21VIR10433-11/2021 | H5N1 | B2 | 2021-10-12 | white-tailed eagle | Estonia | y | 7778754 |
| A/gray_heron/Denmark/24326-1.02/2021 | H5N1 | B2 | 2021-10-28 | gray heron | Denmark | y | 11798577 |
| A/chicken/Netherlands/21037750-001005/2021 | H5N1 | B2 | 2021-10-31 | chicken | Netherlands | n | 5942524 |
| A/greylag_goose/Netherlands/21037497-001/2021 | H5N1 | B2 | 2021-10-19 | greylag goose | Netherlands | y | 6328001 |
| A/swan/Netherlands/21037791-001/2021 | H5N1 | B2 | 2021-11-01 | swan | Netherlands | y | 6328009 |
| A/waterfowl/Netherlands/21037914-006/2021 | H5N1 | B2 | 2021-11-02 | waterfowl | Netherlands | y | 6375134 |
| A/domestic_duck/Poland/H1942-N/2021 | H5N1 | B2 | 2021-11-07 | domestic duck | Poland | n | 6934175 |
| A/Turkey/Sweden/SVA211212SZ0001/FB301013-IP-2/M-2021 | H5N1 | B2 | 2021-12-12 | turkey | Sweden | n | 8338002 |
| A/mute_swan/Czech_Republic/21312/2021 | H5N1 | B2 | 2021-11-01 | mute swan | Czech Republic | y | 6328409 |
| A/swan/France/21P012384/2021 | H5N1 | B2 | 2021-11-08 | swan | France | y | 6590766 |
| A/chicken/Czech_Republic/23404/2021 | H5N1 | B2 | 2021-11-25 | chicken | Czech Republic | n | 8515480 |
| A/barnacle_goose/Denmark/24273-1.02/2021 | H5N1 | B2 | 2021-10-26 | barnacle goose | Denmark | y | 11798568 |
| A/common_buzzard/Denmark/24271-1.02/2021 | H5N1 | B2 | 2021-10-25 | common buzzard | Denmark | y | 11798571 |
| A/Eurasian wigeon/Germany-SH/AI06143/2021 mix2 | H5N1 | B2 | 2021-10-22 | eurasian wigeon | Germany | y | 7753387 |
| A/chicken/Denmark/24357-11/2021 | H5N1 | B2 | 2021-11-02 | chicken | Denmark | n | 11798570 |
| A/turkey/Denmark/24325-25/2021 | H5N1 | B2 | 2021-10-30 | turkey | Denmark | n | 11798580 |
| A/barnacle goose/Germany-SH/AI06145/2021 | H5N1 | B2 | 2021-10-25 | barnacle goose | Germany | y | 7753407 |
| A/white-tailed eagle/Germany-MV/AI05975/2021 | H5N1 | B2 | 2021-10-20 | white-tailed eagle | Germany | y | 7753272 |
| A/domestic duck/Germany-SH/AI06221/2021 | H5N1 | B2 | 2021-10-30 | domestic duck | Germany | n | 11725869 |
| A/turkey/Germany-MV/AI06035/2021 | H5N1 | B2 | 2021-10-20 | turkey | Germany | n | 7753290 |
| A/white stork/Germany-MV/AI05979/2021 | H5N1 | B2 | 2021-10-20 | white stork | Germany | n | 7753329 |
| A/lesser white-fronted goose/Germany-MV/AI05973/2021 | H5N1 | B2 | 2021-10-20 | lesser white-fronted goose | Germany | n | 7753309 |
| A/Wild_goose/Italy/21VIR10193/2021 | H5N1 | B2 | 2021-11-23 | wild goose | Italy | y | 7733611 |
| A/goose/Tyumen/33-52/2021 | H5N1 | B2 | 2021-10-07 | goose | Russian Federation | n | 5463805 |
| A/chicken/Tyumen/33-45/2021 | H5N1 | B2 | 2021-10-07 | chicken | Russian Federation | n | 5463804 |
| A/goose/Tyumen/33-53/2021 | H5N1 | B2 | 2021-10-07 | goose | Russian Federation | n | 5463806 |
| A/grey_heron/Czech_Republic/23608-1K/2021 | H5N1 | B2 | 2021-11-28 | grey heron | Czech Republic | y | 8515482 |
| A/great_egret/Czech_Republic/23609/2021 | H5N1 | B2 | 2021-11-28 | great egret | Czech Republic | y | 8515483 |
| A/mute_swan/Croatia/146/2021 | H5N1 | B2 | 2021-12-29 | mute swan | Croatia | y | 8439476 |
| A/chicken/Italy/IZSLT-122448_21VIR9218-1/2021 | H5N1 | B2 | 2021-10-28 | chicken | Italy | n | 7733644 |
| A/Cygnus_olor/Romania/16381_21VIR10306/2021 | H5N1 | B2 | 2021-11-11 | mute swan | Romania | y | 8440175 |
| A/egret/France/21P013418/2021 | H5N1 | B2 | 2021-12-03 | egret | France | y | 8377254 |
| A/goose/Italy/IZSLT-21VIR10273/2021 | H5N1 | B2 | 2021-11-22 | goose | Italy | n | 7733645 |
| A/chicken/Italy/21VIR10389/2021 | H5N1 | B2 | 2021-11-30 | chicken | Italy | n | 7733599 |
| A/chicken/Netherlands/21038675-001005/2021 | H5N1 | B2 | 2021-11-14 | chicken | Netherlands | n | 6328036 |
| A/bean_goose/Sweden/SVA211111SZ0372/FB004482/2021 | H5N1 | B2 | 2021-11-05 | bean goose | Sweden | y | 7049600 |
| A/mute_swan/Croatia/100/2021 | H5N1 | B2 | 2021-11-12 | mute swan | Croatia | y | 6507374 |
| A/goose/Croatia/107/2021 | H5N1 | B2 | 2021-11-18 | goose | Croatia | n | 7570634 |
| A/greylag_goose_/Sweden/SVA211118SZ0354/FB004497/I-2021 | H5N1 | B2 | 2021-11-17 | greylag goose | Sweden | y | 7055384 |
| A/grey_heron/Croatia/132/2021 | H5N1 | B2 | 2021-12-08 | grey heron | Croatia | y | 7892492 |
| A/domestic_goose/Poland/H1931-T1/2021 | H5N1 | B2 | 2021-11-03 | domestic goose | Poland | n | 6931008 |
| A/white-tailed_eagle/Sweden/SVA211201SZ0380/FB004721/M-2021 | H5N1 | B2 | 2021-11-26 | white-tailed eagle | Sweden | y | 7635907 |
| A/Red_fox/Estonia/TA2126820_21VIR10433-13/2021 | H5N1 | B2 | 2021-11-08 | Red fox | Estonia | y | 7778880 |
| A/buzzard/Germany-SH/AI07099/2021 | H5N1 | B2 | 2021-11-09 | buzzard | Germany | y | 11725994 |
| A/Eurasian_wigeon/Italy/21VIR8919-3/2021 | H5N1 | B2 | 2021-10-28 | eurasian wigeon | Italy | y | 7733585 |
| A/greylag_goose_/Sweden/SVA211111SZ0376/FB004497/M-2021 | H5N1 | B2 | 2021-11-08 | greylag goose | Sweden | y | 7054529 |
| A/gadwall/Croatia/108/2021 | H5N1 | B2 | 2021-11-22 | gadwall | Croatia | y | 8568483 |
