# Supplementary material for: Has Epizootic Become Enzootic? Evidence for a Fundamental Change in the Infection Dynamics of Highly Pathogenic Avian Influenza in Europe, 2021
Source: mBio. 2022 Jun 21;13(4):e00609-22. doi: 10.1128/mbio.00609-22 (PMC9426456; doi:10.1128/mbio.00609-22)
Supplement: TEXT S1 [file mbio.00609-22-s0005.docx]

**Supplemental:**

**Material and Methods**

**Samples**

Sampling material from wild birds and poultry originated from passive monitoring activities practised in the different EU member states and the UK. Material that tested positive, by real-time RT-qPCR and pathotype analyses, for HPAI viruses was selected for sequencing.

**Sequencing**

Several techniques of next generation-based sequencing were employed. In most cases, sequences were established directly from clinical material. Details are available from the authors.

**Phylogenetic analyses**

Maximum likelihood (ML) analyses were carried out by use of the IQ-tree software, version 2.1.3 (1). The best-fit substitution model FLU+R2+F was selected by IQ Tree’s ModelFinder and the Bayesian inference criterion (2). SH-aLRT and ultrafast bootstrap-equivalent analyses, each of 10000 iterations, were used to assess nodal robustness of the consensus trees (3). Results were viewed and edited using the FigTree v1.4.2 (<http://tree.bio.ed.ac.uk/software/figtree/>) and Inkscape 0.92 ([https://inkscape.org](https://inkscape.org/)) software. Maximum likelihood (ML) trees were also generated separately for each genome segment with RAxML (4) utilizing the model GTR GAMMA with rapid bootstrapping and search for the best scoring maximum likelihood tree together with 1000 bootstrap replicates. For genotypic analyses the alignments were curated to identify outliers, remove duplicated, identical and incomplete sequences. Coding sequences of this selection were concatenated, aligned (Geneious Prime Biomatters, New Zealand) and ML trees generated with RAxML. For visualization the resulting tree was plotted in polar layout with proportional transformed branches. Branches were colored according to the genotype. For time-scaled trees of the HA sequences of all genotypes dataset were expanded to include precursor H5 clade 2.3.4.4 viruses and manually curated and annotated using Geneious Prime 2021.0.1 (Biomatters) to exclude identical viral sequences. Time-scaled maximum clade credibility (MCC) analysis (date of sample collection) was calculated with BEAST (V1.10.4) software package (5) using a GTR GAMMA substitution model, an uncorrelated relaxed clock with a lognormal distribution and coalescent constant population tree models. Chain length was set to 50 million iterations and convergence checked via Tracer (V1.7.1). Time-scaled summary maximum clade credibility trees (MCC) with 10% for the post burn-in posterior were created using TreeAnnotator (V1.10.4) and visualised with FigTree (V1.4.4).

**References for M&M**

1. Minh BQ, Schmidt HA, Chernomor O, Schrempf O, Woodhams MD, von Haeseler A, Lanfear R. 2020. IQ-TREE 2: New models and efficient methods for phylogenetic inference in the genomic era. *Mol Biol Evol* 37: 1530-1534.
2. Kalyaanamoorthy S, Minh BQ, Wong TKF, von Haeseler A, Jermiin LS. 2017. ModelFinder: Fast model selection for accurate phylogenetic estimates. *Nat Methods*14: 587-589.
3. Hoang DT, Chernomor O, von Haeseler A, Minh BQ, Vinh LS. 2918. UFBoot2: Improving the ultrafast bootstrap approximation. *Mol Biol Evol* 35: 518–522.
4. Stamatakis A. 2014. RAxML version 8: a tool for phylogenetic analysis and post-analysis of large phylogenies. *Bioinformatics* 30: 1312-3.
5. Suchard MA, Lemey P, Baele G, Ayres DL, Drummond AJ, Rambaut A. 2018. Bayesian phylogenetic and phylodynamic data integration using BEAST 1.10. *Virus Evol* 4: vey016.
